# Unusual gastrointestinal manifestations of COVID-19: two case reports 

**Published:** 2020

**Authors:** Amir Hossein Hassani, Alireza Beheshti, Faezeh Almasi, Pardis Ketabi Moghaddam, Mohammadreza Azizi, Shabnam Shahrokh

**Affiliations:** *Gastroenterology and Liver Diseases Research Center, Research Institute for Gastroenterology and Liver Diseases, Shahid Beheshti University of Medical Sciences, Tehran, Iran *

**Keywords:** Case reports, Gastrointestinal manifestations, COVID-19

## Abstract

As of December 2019, a new strain of coronavirus named severe acute respiratory syndrome coronavirus-2 (SARS-CoV-2) was discovered in Wuhan, China, following an epidemic of a fast-spreading viral respiratory disease, later called Coronavirus Disease 2019 (COVID-19), which then lead to the present pandemic the world has come to know. Patients who tested positive for COVID-19 are mostly asymptomatic or present with mild self-limiting symptoms. While GI symptoms occur with less prevalence, they are increasingly being reported. A diagnosis of Covid-19 has increased dramatically in patients presenting with gastrointestinal symptoms suggesting that GI symptoms should be taken into serious consideration with patient diagnosis.

**Case 1:** A 65-year-old man presented to the hospital emergency room with abdominal pain, Murphy's sign and chills without fever, subsequently diagnosed as acute acalculous cholecystitis with a positive COVID-19 rRT-PCR.

**Case 2:** A 78-year-old woman presented to the hospital emergency room complaining of severe positional epigastric pain precipitated by lying supine, chills with no fever, being later diagnosed as acute pancreatitis and a positive COVID-19 rRT-PCR. It has become evident that the ACE2 receptor plays a significant role as the entry site into human cells for the virus. This receptor is generally expressed in respiratory cells, as well as the gastrointestinal tract, corresponding with extrapulmonary manifestations of COVID-19. Studies concluded that the origin of gastrointestinal symptoms could be caused by the interaction of the SARS-CoV-2 virus with cells through the ACE2 receptor. The findings of the present study support this theory, as both patients presented with symptoms regarding tissues with high ACE2 expression.

## Introduction

 The widespread disease of COVID-19, affecting more than 29 million people across 216 countries,(6) seems to have originated from the Hubei province, Wuhan, China, in December 2019, and of which WHO declared this a pandemic on March 11, 2020.(7) The initial studies revealed general and respiratory symptoms such as fever (98.6%), dry cough (59.4%), malaise (69.6%), and dyspnea (31.2%) to be the most common manifestations of the disease at the time.(3) However, as time passed and the COVID-19 virus became widespread, atypical symptoms began to attract the attention of physicians. Studies began to focus on symptoms such as GI involvement, cardiac disease, and renal impairment. Nevertheless, COVID-19 is still considered a novel disease. Although the knowledge behind the pathophysiology and clinical manifestations is expanding rapidly, it might be too early to draw a line between typical and atypical symptoms. As it can be seen from our mentioned cases, patients can demonstrate uncommon symptoms such as severe abdominal pain at the time of presentation, which might even be the symptom of a complication caused by COVID-19 and not the virus itself. For instance, the second case with acute viral pancreatitis could be one of many complications encountered with this disease. At the time of admitting these patients, the guidelines for inspecting patients suspicious of COVID-19 consisted of acquiring chest CT scans, rRT-PCR, or both.(8) As of March 22, 2020, the American College of Radiology does not recommend using a chest CT scan to screen patients.(9) However, it can be used in conjunction with rRT-PCR to diagnose patients highly suspicious of COVID-19 despite a negative rRT-PCR test or in case of inadequate or insufficient rRT-PCR equipment, given it is more sensitive than the rRT-PCR test.(8) The body of knowledge is growing regarding the pathophysiology of this disease. It has become evident that the ACE2 receptor plays a major role as the site of entry into human cells for the virus. This receptor is mainly expressed in respiratory cells. However, it is present in other organs such as the heart, kidneys, and the GI tract, specifically the gastric, duodenum, hepatic, and biliary epithelium and pancreatic islets. The presence of these receptors corresponds with the extrapulmonary manifestations of COIVID-19. The ACE2 receptor in the GI tract is a regulator of inflammatory responses. From there, studies have concluded that the origin of gastrointestinal symptoms could have one root in the interaction of the COVID-19 virus with cells through the ACE2 receptor.(10) Research is being conducted on this matter and there is much progress to be made in the future. The first studies published on COVID-19 mostly consisted of fever and respiratory symptoms as the primary manifestations of the disease leaving space for disregarding other symptoms. Studies investigating the prevalence of GI symptoms in COVID-19 patients have increased over time, which has led to a broader understanding of the various manifestations of this disease. Jin, X. et al. conducted a study in the Zhejiang province, China, reporting gastrointestinal symptoms to be 11.4 %, much greater than initially described in earlier studies in Wuhan.(5) This study, and many others, have not only illustrated the importance of detecting these symptoms but have also raised suspicions on GI manifestations as part of the COVID-19 clinical presentations; sometimes even as the only one.The first case discussed in this study complained of epigastric pain, mostly in the RUQ, later diagnosed as acute a calculus cholecystitis, which to the best of our knowledge is the first case to be reported from COVID-19 patients in Iran. Few studies have been published reporting similar cases in COVID-19 patients, and the evidence behind a relationship between SARS-CoV-2 and biliary involvement is scarce, even as a complication of COVID-19. Although the pathogenesis mentioned above regarding the ACE2 receptor could be at play, it is not well understood projecting that there is grounds for further investigations. The second case we discussed in this study initially presented with symptoms suggestive of acute pancreatitis, one of the few cases reported in Iran. In light of the medical history and lack of risk factors, SARS-CoV-2 infection might have been the cause. Several case reports of COVID-19 have been published that initially presented with acute pancreatitis, and other studies have revealed COVID-19 patients developing acute pancreatitis during hospitalization. In keeping with the already known pathophysiology of the disease, the interaction of SARS-CoV-2 with pancreatic islet cells by binding to the ACE2 receptor could be the etiology behind this phenomenon.(11, 12) Nonetheless, further research is required to reach such conclusions considering the apparent limitations of this study. Our understanding of clinical manifestations of COVID-19 is growing day by day and new studies are being conducted, and published, informing us about new ways of dealing with this disease. Until reaching a worldwide consensus, it is of utmost importance to take uncommon symptoms into account and be aware of the unusual ways COVID-19 can present, as these cases above have illustrated, to prevent further spread and identify patients earlier, provide them with better health care, and potentially save their lives.

## Case Report

**Case 1**

**Figure 1:**
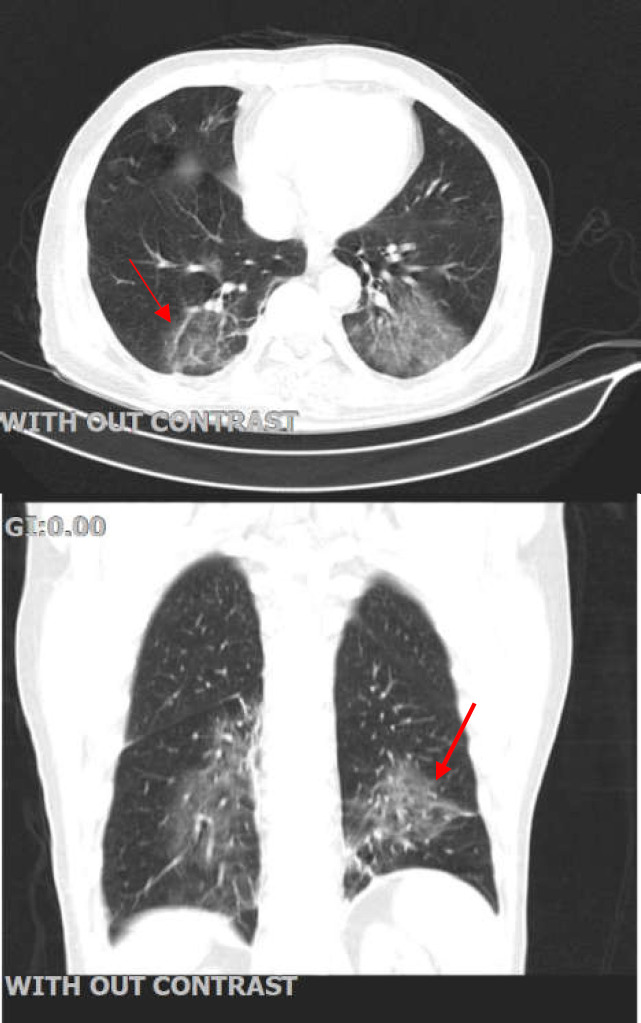
Chest CT scan of the patient. On the chest CT scan, bilateral infiltrations are evident

On February 23, 2020, a 65-year-old man presented to the emergency room of Taleghani Hospital with abdominal pain and intermittent shaking chills without fever. The patient stated the symptoms had an abrupt onset followed by severe epigastric pain also reporting two episodes of non-biliary vomiting. The patient reported a 20-year history of hypertension and ischemic heart disease, for which he had undergone coronary bypass graft surgery 15 years prior. His medications included Metoral 50mg twice daily, Atorvastatin 20mg daily, and Losartan 50mg twice daily. He did not smoke, drink alcohol, or use illicit drugs, and he had no known history of allergies. The patient exhibited a person being ill. He had a body temperature of 36.8°C, a pulse rate of 90 bpm, a blood pressure of 122/75 mm Hg, a respiratory rate of 14 per minute, and an oxygen saturation of 98% in room air. On physical examination, the abdomen was soft with normal bowel sounds but with considerable tenderness on palpation of the right upper quadrant region indicating Murphy’s sign. Apart from a CABG scar on the sternum and mild pallor, no other findings were evident during the physical examinations. After the initial evaluation, an abdominal ultrasonography was performed reporting an increased gallbladder wall thickness (4.5mm), normal CBD diameter without apparent cholelithiasis or sludge formation. Further investigations with EUS revealed no stones or lesions that might be the cause of the cholecystitis. With the ultrasonography, EUS findings and clinical status in consideration, we admitted the patient to the gastroenterology ward with a diagnosis of acute acalculus cholecystitis. Having the recent COVID-19 outbreak and its unusual presentations in mind, we transferred the patient to the airborne-isolation unit, collected, and sent nasopharyngeal and oropharyngeal swab specimens for real-time reverse-transcriptase–polymerase-chain-reaction (rRT-PCR) assay with suspicion of COVID-19. A chest CT scan was also obtained. The CT scan study revealed patchy peripheral ground glass infiltrations in both lungs, particularly in the lower lobes, suggestive of COVID-19 (Figure 1).

Given the CT scan findings, we initiated treatment for COVID-19 with Favipiravir (1600 mg loading dose orally followed by 600 mg orally twice daily) in conjunction with supportive treatment, including intravenous crystalloid resuscitation and analgesics. Laboratory results of the first day of admission showed leukopenia (980 lymphocytes per µL) and an elevated CRP (15 mg/L). In addition, alkaline phosphatase (708 U/L), alanine aminotransferase (258 iU/L), and aspartate aminotransferase (157 iU/L) were all elevated. On day 2 of admission, the patient developed a mild fever, was unresponsive to antipyretics, and mentioned that he had an episode of transient dyspnea later that day. Otherwise, vital signs and physical examinations were normal. Later on that the same day, the patient's nasopharyngeal and oropharyngeal swab test was confirmed positive by rRT-PCR for COVID-19. From day 3 through 5 of hospitalization, the patient's clinical condition remained relatively stable, however experiencing one more episode of dyspnea with an O2 saturation of 90% in room air. Otherwise, the patient voiced no complaints. Following treatment for COVID-19, biliary pain and fever resolved during this period and his bowel movements were regular. 

On day 3 of hospitalization, the creatinine level rose (from 1.2 to 2.3 mg/dL) but normalized the very next day. On day 5 of admission, the patient was afebrile, dyspnea had resolved, and reported no abdominal pain. Lung auscultation was normal, and other physical examinations were unremarkable. With remission of the disease, the patient was discharged from the hospital, and on a one-week follow up there were no complaints.

**Case 2**

**Figure 2:**
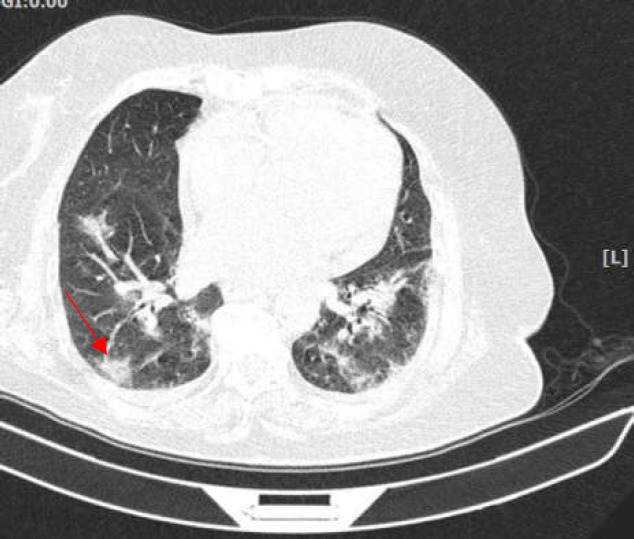
Chest CT scan of the patient. On the chest CT scan, bilateral infiltrations are evident

On March 3, 2020, a 78-year-old woman presented to the Taleghani hospital emergency room complaining of severe positional epigastric pain, precipitated by lying supine, and chills with no fever, also mentioning a few episodes of nausea and vomiting. The patient had a history of hypertension and ischemic heart disease. She was under treatment with Prazosin 1 mg capsule twice daily, Valsartan 80 mg tablet twice daily, Clopidogrel 75 mg tablet once daily, Aspirin 80 mg tablet once daily, and Atorvastatin 40 mg tablet once daily. The patient was a non-smoker, had no history of alcohol consumption, and no known allergy history. The patient showed signs of being an ill person. The body temperature was 37°C, pulse rate of 80 bpm, blood pressure 130/80 mm Hg, respiratory rate of 12 per minute, and an oxygen saturation of 94% breathing ambient air. On physical examination, the abdomen was soft with normal bowel sounds. Severe tenderness on palpation of the epigastric region radiating to the back was apparent. No other findings were evident during the physical examinations. Considering the typical acute pancreatitis presentation of the patient, we obtained laboratory tests including Amylase and Lipase. Initial laboratory results revealed an increased Amylase (185 iU/L) and Lipase (230 iU/L). The patient was admitted to the gastroenterology ward with acute pancreatitis diagnosis. Other notable laboratory results of the first day are as follows: mild thrombocytopenia (149000/µL), BUN (22 mg/dL), creatinine (1.4 mg/dL), alanine aminotransferase (35 iU/L), and aspartate aminotransferase (40 iU/L). On the first day of admission, to elucidate the underlying etiology, we conducted further assessments, including: Liver and bile ducts ultrasound study, a lipid profile, and serum electrolyte levels. The ultrasound study reported normal liver parenchyma with normal gallbladder and CBD diameter. No gallstones were observed with heterogeneity of pancreas parenchyma and increased pancreas volume in favor of acute pancreatitis. Lipid profile and electrolyte levels were unremarkable. No cause for pancreatitis was found during the evaluation. On day 2 of admission, the patient reported a dry cough from the previous night disturbing sleep. Given the COVID-19 pandemic, we transferred the patient to the airborne isolation unit and requested a chest CT scan. We found patchy peripheral ground glass infiltrations in both lungs on the chest CT scan suggestive of COVID-19 (Figure 2). Oropharyngeal and nasopharyngeal swab specimens were collected and sent to be tested for COVID-19 with real-time reverse-transcriptase–polymerase-chain-reaction (rRT-PCR) assay. 

With high suspicion of COVID-19, we initiated supportive treatment consisting of supplemental oxygen, delivered by nasal cannula at 2 liters per minute, intravenous crystalloid fluid resuscitation, analgesics, and a bowel rest. On the following day, the patient did not improve upon the initiation of supportive care. Dry cough and abdominal pain persisted, and the patient developed a fever unresponsive to antipyretics. On day 4 of admission, the patient became hypoxic with an O2 saturation of 89% with a nasal cannula. Following the respiratory distress progression, the patient was intubated and was admitted to the Intensive Care Unit (ICU) for mechanical ventilation. On the same day, the patient's nasopharyngeal and oropharyngeal swab test was confirmed positive by rRT-PCR for COVID-19. On that note, treatment was initiated with intravenous Remdesivir (200 mg IV loading dose followed by 100 mg IV daily) and Interferon Beta-1b (8 million international units SQ, q.o.d.). A follow-up chest and abdominal CT scan was obtained, which revealed a remarkable progression of bilateral patchy ground-glass opacities. Evidence of necrotizing pancreatitis was also observed. Over the course of ICU admission, the patient's condition worsened. Moreover, laboratory results showed a significant rise in Amylase (1200 iU/L) and Lipase (1450 iU/L) levels. The patient developed signs of multiple organ failure on the following days, primarily affecting the kidneys, becoming oliguric, followed by hypotension and bradycardia. On March 9, 2020 (day 7 of admission), the patient started to deteriorate, becoming hemodynamically unstable, resulting in a cardiac arrest, which CPR failed to resuscitate.

## Discussion

As of December 2019, a new strain of coronavirus named severe acute respiratory syndrome coronavirus-2 (SARS-CoV-2) was discovered in Wuhan, China, following an epidemic of a fast-spreading viral respiratory disease, later called Coronavirus Disease 2019 (COVID-19), which subsequently lead to a pandemic.(1) The vast global spread of this disease has given rise to a new cause of mortality worldwide, alarming nations and causing distress upon multiple aspects of the healthcare system. Due to its broad spectrum of unspecific clinical presentations and lack of sensitive tests, especially in the early stages of the disease, reaching a diagnosis based upon clinical findings has presented an enormous challenge.(2) Increasingly reported cases of patients who tested positive for COVID-19 mostly present asymptomatic or self-limiting symptoms varying from fever, dry cough, dyspnea, and fatigue, to less prevalent ones such as renal impairment and GI involvement.(3) However, a notable percentage of patients advance to moderate to severe forms of the disease, developing complications such as acute respiratory distress syndrome, sepsis, septic shock, or even death, making an early diagnosis of substantial importance.(4) Concluding a diagnosis of Covid-19 has increased dramatically in patients presenting with gastrointestinal symptoms such as loss of appetite, nausea and vomiting, diarrhea, and abdominal pain before developing pulmonary manifestations, suggesting that GI symptoms should be taken into serious consideration.(5)Owing to the lack of understanding of the extrapulmonary involvements in COVID-19, reporting such cases may provide further insight into the pathogenesis of the yet novel disease and assist other clinicians in managing similar conditions when encountered, potentially reaching a consensus when approaching certain patients. Here we provide two patient cases diagnosed as COVID-19 who initially presented with GI symptoms.

## Conflict of interests

The authors declare that they have no conflict of interest.
